# Leveraging ChatGPT for thematic analysis of medical best practice advisory data

**DOI:** 10.1093/jamiaopen/ooaf126

**Published:** 2025-10-27

**Authors:** Yejin Jeong, Margaret Smith, Robert J Gallo, Lisa Marie Knowlton, Steven Lin, Lisa Shieh

**Affiliations:** Stanford Healthcare AI Applied Research Team, Division of Primary Care and Population Health, Stanford University School of Medicine, Stanford, CA 94063, United States; Stanford Healthcare AI Applied Research Team, Division of Primary Care and Population Health, Stanford University School of Medicine, Stanford, CA 94063, United States; Center for Innovation to Implementation, VA Palo Alto Health Care System, Menlo Park, CA 94025, United States; Department of Health Policy, Stanford University, Stanford, CA 94305, United States; Section of Acute Care Surgery, Department of Surgery, Stanford University, Stanford, CA 94304, United States; Stanford Healthcare AI Applied Research Team, Division of Primary Care and Population Health, Stanford University School of Medicine, Stanford, CA 94063, United States; Department of Medicine, Stanford University School of Medicine, Stanford, CA 94305, United States; Department of Medicine, Stanford University School of Medicine, Stanford, CA 94305, United States

**Keywords:** artificial intelligence, large language models, thematic analysis, qualitative research, informatics, prompt engineering

## Abstract

**Objectives:**

To evaluate ChatGPT’s ability to perform thematic analysis of medical Best Practice Advisory (BPA) free-text comments and identify prompt engineering strategies that optimize performance.

**Materials and Methods:**

We analyzed 778 BPA comments from a pilot AI-enabled clinical deterioration intervention at Stanford Hospital, categorized as reasons for deterioration (Category 1) and care team actions (Category 2). Prompt engineering strategies (role, context specification, stepwise instructions, few-shot prompting, and dialogue-based calibration) were tested on a 20% random subsample to determine the best-performing prompt. Using that prompt, ChatGPT conducted deductive coding on the full dataset followed by inductive analysis. Agreement with human coding was assessed as inter-rater reliability (IRR) using Cohen’s Kappa (κ).

**Results:**

With structured prompts and calibration, ChatGPT achieved substantial agreement with human coding (κ = 0.76 for Category 1; κ = 0.78 for Category 2). Baseline agreement was higher for Category 1 than Category 2, reflecting differences in comment type and complexity, but calibration improved both. Inductive analysis yielded 9 themes, with ChatGPT-generated themes closely aligning with human coding.

**Discussion:**

ChatGPT can accelerate qualitative analysis, but its rigor depends heavily on prompt engineering. Key strategies included role and context specification, pulse-check calibration, and safeguard techniques, which enhanced reliability and reproducibility.

**Conclusion:**

This study demonstrates the feasibility of ChatGPT-assisted thematic analysis and introduces a structured approach for applying LLMs to qualitative analysis of clinical free-text data, underscoring prompt engineering as a methodological lever.

## Background and significance

Manual coding of clinical free-text remains a major barrier to scalable qualitative analysis in healthcare due to its labor-intensive nature.^1^ Large language models (LLMs) have been explored as tools to support tasks such as coding and theme identification.^2–8^ Although concerns remain about whether LLMs can meet the rigorous standards of qualitative research, recent work shows that, in some cases, they can achieve performance comparable to human coders and provide additional interpretive depth in thematic summarization.^9^

Most evaluations of LLM-assisted qualitative analysis to date have focused on subjective sources such as interviews or focus groups, which are inherently prone to variability in interpretation.^10–13^ In contrast, clinical free-text generated in electronic health records (EHRs) is often more objective and task-oriented, yet remains underutilized for qualitative insights due to the burden of manual coding. One example is the Best Practice Advisory (BPA), a common form of clinical decision support (CDS) alert in the EHR. A BPA typically appears as a real-time pop-up prompt to guide providers—for example, suggesting additional labs if sepsis criteria are met—and often includes an option for providers to enter free-text comments. These comments represent a unique yet largely underexplored source of qualitative data.

In this study, we address the methodological question of how to design effective prompts for AI-assisted thematic analysis in this context. Using provider free-text comments from a pilot AI-enabled clinical deterioration intervention at Stanford Hospital,^14^ we systematically tested prompt engineering strategies—including role specification, contextual priming, stepwise prompting, and few-shot prompting—and evaluated their impact on coding reliability. By documenting how these prompt refinements influenced model performance, we aim to inform best practices for applying LLMs to healthcare free-text analysis.

## Objective

To evaluate ChatGPT’s ability to perform thematic analysis of BPA free-text comments and to identify strategies for optimizing LLM performance in qualitative analysis.

Specifically, we asked:

1. How effectively can ChatGPT identify and classify recurring themes in BPA free-text comments related to clinical decision-making and provider communication?
2. What prompt engineering strategies optimize ChatGPT’s performance in qualitative analysis for this type of dataset?

## Materials and methods

### Data sources

From January 2021 to November 2022, Stanford Hospital piloted an AI–driven intervention to detect clinical deterioration on 4 medical units, using the Epic Deterioration Index (EDI), a numeric score designed to predict patients at risk of deterioration.^15^ When a patient’s EDI score exceeded 65, a Best Practice Advisory (BPA) alert was triggered, prompting a collaborative huddle of physicians and nurses to assess the patient and document their evaluation. Documentation captured both (1) reasons for deterioration and (2) corresponding care team actions. Providers could select predefined checkbox options (eg, “cardiac system,” “infection,” “new orders,” “continue to monitor”) or enter free-text responses under “Other.”

During the pilot, 778 free-text entries were recorded, providing rich, unstructured data on how providers engaged with the tool in real-world practice. Example comments included “Low blood pressure, O_2_ requirement” (reason for deterioration) and “Comfort care initiated after discussion with family” (care team action). The final dataset included comments ranging from 9 to 512 characters, with an average length of 106 characters. This study used de-identified free-text comments generated during a previously reported quality improvement intervention at Stanford Hospital. As a secondary methodological evaluation of these data, individual provider consent was not required.

### Study design

**Figure 1.**
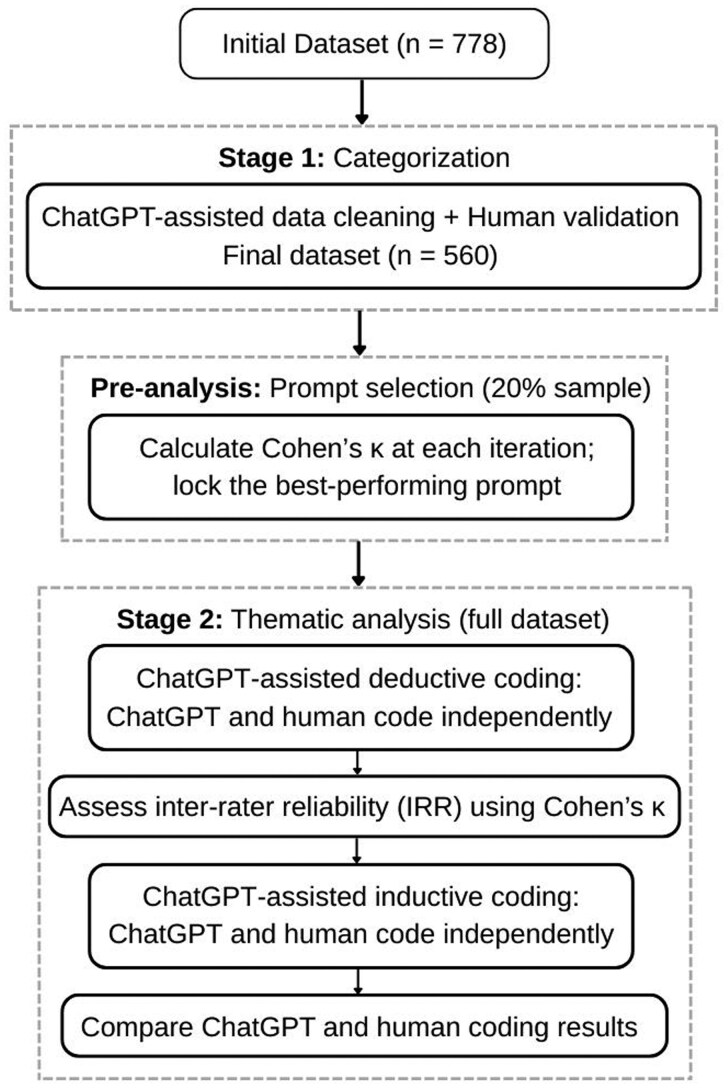
Analysis workflow. Stage 1 involved data cleaning with human validation to generate the final dataset. A pre-analysis step on a 20% random sample was used to identify and lock the best-performing prompt. In Stage 2, this prompt was applied to the full dataset for deductive coding, followed by inductive coding to identify additional themes.

Our analysis proceeded in 2 stages (Figure 1): (1) data categorization and (2) thematic analysis. Prior to full analysis, we conducted a pre-analysis prompt selection on a subsample to identify the best-performing prompt (see “Prompt Engineering”).

#### Stage 1: categorization

The dataset consisted of 778 free-text comments that were intermingled across 2 types. To prepare for analysis, we used ChatGPT to assist in categorizing them into 2 groups: (1) reasons for deterioration and (2) care team actions. This was performed using the Stanford Health Care-School of Medicine (SHC-SoM) Secure GPT-3.5 (beta), which provides access to the model in a HIPAA-compliant institutional environment. Three human reviewers validated the classifications, with discordant cases adjudicated by clinical reviewers.

#### Stage 2: thematic analysis

Within each category, we applied both deductive and inductive coding. Deductive coding used a clinician-developed codebook with an “Other” category for comments that did not fit any predefined codes (see Tables S1 and S2). ChatGPT and a human coder independently coded the data in a blinded manner, with the model treated as an additional coder. Inter-rater reliability (IRR) was assessed at the theme level using percent agreement and Cohen’s κ. Inductive coding was then applied to comments remaining in the “Other” category to identify emerging themes beyond the codebook, and GPT- and human-generated themes were compared to evaluate alignment and divergence.

For this stage, we used the ChatGPT-5 web interface (OpenAI) after verifying that all entries are fully de-identified and free of protected health information (PHI). We selected GPT-5 given its reported improvements in reasoning and instruction adherence, and reduced hallucination compared with earlier models.^16^ Each coding prompt was run in a new chat session with memory disabled, ensuring that prior outputs did not influence subsequent responses.

### Prompt engineering

Prompt strategies were iteratively refined to optimize performance for deductive coding, proceeding in 3 phases (A-C), drawing on techniques from the NLP literature, including role specification, contextual priming, stepwise instructions, and few-shot prompting.^17–23^

#### Phase A: pre-analysis prompt selection

We tested a series of prompt variations on a 20% random subsample. An overview of strategies is presented in Table 1. Agreement with human coding was assessed as inter-rater reliability (IRR) using Cohen’s κ after each iteration, and the-best performing prompt was “locked” for subsequent full analyses.

**Table 1.** Overview of prompt strategies explored in this study.

Prompt strategy	Description
Baseline (zero-shot)	Model asked to classify each comment into one of the predefined categories.
Role specification + Contextual priming	Model was instructed to act as a clinical qualitative coding expert analyzing provider BPA free-text comments in a hospital deterioration alert context.
Stepwise instructions (chain-of-thought)	Added structured reasoning steps (eg, read comments, extract clinical cues, map to a system, decide final category) to encourage deliberation beyond simple keyword matching.
Few-shot prompting	Provided example comments with corresponding correct categories before asking the model to classify new comments.

#### Phase B: human-model calibration

In addition to single-pass prompting, we explored a dialogue-based calibration step inspired by standard practices in qualitative research where human coders pilot a subset before coding the full dataset. In this approach, the locked prompt was first applied to 20% of the data (“pulse-check”). Discordant cases were then revisited in a dialogue, during which the model articulated its rationale and both model and human coder refined their reasoning.

#### Phase C: full dataset application

Following calibration, the locked prompts were applied to the full dataset (Category 1: *n* = 357; Category 2: *n* = 203), and inter-rater reliability was assessed using Cohen’s κ.

## Results

### Data preparation and categorization

During data cleaning, ChatGPT demonstrated high accuracy in categorizing BPA free-text comments. Of the 778 entries, 153 were excluded as non-informative. Among the remaining 625, ChatGPT’s classifications were reviewed by 3 human reviewers, who judged 578 (93%) to be correct. The remaining 47 (7%) discordant cases underwent secondary clinical review, which confirmed 37 true misclassifications and reinstated 5 comments initially flagged in error. Comments that did not clearly fit either category were excluded. The final dataset included 560 comments: 357 related to reasons for deterioration (Category 1) and 203 describing care team actions (Category 2).

### Prompt engineering phase A: pre-analysis prompt selection

To account for run-to-run variability, each prompt strategy was tested 3 times on the same 20% subsample (*n* = 70 for Category 1; *n* = 40 for Category 2), with mean percent agreement and Cohen’s κ reported (Tables 2 and 3).

**Table 2.** Iterative prompt refinement on Category 1 subsample (*n* = 70).

Iteration	Prompt strategy	Matched N (%)	Cohen’s κ (range)	Agreement
I0	Baseline	53/70 (75.7%)	0.67 (0.66-0.68)	Substantial
I1	Baseline + [Role + Context]	58/70 (82.9%)	0.75 (0.74-0.77)	Substantial
I2	Stepwise instructions	52/70 (74.3%)	0.64	Substantial
I3	Stepwise + [Role + Context]	56/70 (80.0%)	0.71 (0.68-0.76)	Substantial
I4	Few-shot prompting	56/70 (80.0%)	0.72	Substantial
I5	Few-shot + Stepwise	56/70 (80.0%)	0.72 (0.70-0.73)	Substantial
I6	Few + Step + [Role + Context]	58/70 (82.9%)	0.76 (0.74-0.80)	Substantial

**Table 3.** Iterative prompt refinement on Category 2 subsample (*n* = 40).

Iteration	Prompt strategy	Matched N (%)	Cohen’s κ (range)	Agreement
I0	Baseline	19/40 (47.5%)	0.42 (0.38-0.44)	Moderate
I1	Baseline + [Role + Context]	31/40 (72.5%)	0.73 (0.69-0.78)	Substantial
I2	Stepwise instructions	22/40 (55.0%)	0.49 (0.46-0.50)	Moderate
I3	Stepwise + [Role + Context]	22/40 (55.0%)	0.48 (0.43-0.52)	Moderate
I4	Few-shot prompting	26/40 (65.0%)	0.57 (0.57-0.58)	Moderate
I5	Few-shot + Stepwise	26/40 (65.0%)	0.59 (0.58-0.60)	Moderate
I6	Few + Step + [Role + Context]	27/40 (67.5%)	0.60 (0.58-0.63)	Substantial

For Category 1, the baseline prompt (I0) already achieved substantial agreement (76%, κ = 0.67). Adding role and context (I1) improved performance to 83% (κ = 0.75), with the best overall performance observed when all strategies were combined (I6: 83%, κ = 0.76). Stepwise instructions alone (I2) reduced performance (74%, κ = 0.64), though combining them with role/context (I3) partially recovered agreement (80%, κ = 0.71). Few-shot prompting (I4-I5) provided modest gains (80%, κ = 0.72). Overall, Category 1 results suggest that although the data was free text, their constrained clinical language and predictable phrasing supported strong baseline performance, with layered strategies offering only incremental improvements.

In contrast, Category 2 showed much weaker baseline performance (48%, κ = 0.42, moderate). Here, prompt refinements made a much larger difference: role + context (I1) yielded the greatest improvement, raising agreement to 73% (κ = 0.73, substantial). Few-shot prompting (I4, I5) provided modest gains (65%, κ = 0.57-0.59), while stepwise reasoning had limited benefit (κ = 0.48-0.49). The full combination (I6) reached 68% agreement (κ = 0.60, substantial), though it did not surpass the role + context strategy.

Together, these patterns suggest that dataset characteristics shaped the relative benefits of prompt refinements, while the addition of role and context consistently produced the greatest gains (see final prompts in Table S3).

### Prompt engineering phase B: human-model calibration

We next tested whether dialogue-based calibration could enhance reliability. The locked prompt for each category (I6 for Category 1; I1 for Category 2) was first applied to a 20% random subsample, and discordant cases were revisited in dialogue until reasoning was aligned (see Table S4). This calibration step improved IRR from κ = 0.71 to κ = 0.76 for Category 1 and from κ = 0.58 to κ = 0.78 for Category 2. The pulse-check thus acted as an early safeguard against systematic misclassification and provided transparency into the model’s reasoning before scaling to the full dataset.

### Prompt engineering phase C: full dataset application

Finally, the calibrated prompts were applied to the entire dataset. Agreement with the human coder was substantial—82% (κ = 0.76) for Category 1 (*n* = 357) and 83% (κ = 0.78) for Category 2 (*n* = 203). These represent the final performance metrics reported in this study.

### Deductive and inductive thematic coding

In Category 1 (reasons for deterioration), most free-text comments mapped to existing categories in the codebook: Respiratory (*n* = 143), Mental/Neuro (*n* = 68), Cardiac (*n* = 54), Infection (*n* = 33), and Swallowing impairment (*n* = 5). A subset of 54 comments fell into “Other” and was subsequently analyzed inductively (see Table S5 for the prompt used). ChatGPT generated 5 themes—System Alert, Lab Abnormality, Procedure/Medication, Other Clinical Concern, and Error—which broadly overlapped with the 3 human-derived themes. Differences were primarily in labeling and granularity than divergence in meaning. Detailed comparisons are provided in Table S6.

In Category 2 (care team actions), fewer comments mapped deductively to established categories: new orders (*n* = 34), continue to monitor (*n* = 22), provide read back (*n* = 17), no new orders (*n* = 15), goals of care discussion (*n* = 15), critical care response nurse consult (*n* = 8), ICU team consult (*n* = 4), new consult (*n* = 3), family meeting (*n* = 2), and transfer to higher level of care (n = 2). Of the remaining 87 comments coded as “Other,” inductive analysis by ChatGPT produced 4 themes: Notify Team, Action/Orders Request, Escalation Request, and Data Correction. These corresponded closely to the 5 human-derived themes, with differences mainly in naming or grouping (eg, ChatGPT combined “Guidance” and “Order Request” into a single category, “Action/Orders Request”) rather than fundamental disagreement.

Across both categories, ChatGPT-generated themes aligned closely with human coding. While the model did not surface entirely novel categories, it produced consistent, clinically intuitive labels and demonstrated reliable thematic coding results. Figures S1 and S2 illustrate visual comparisons of theme distributions before and after ChatGPT-assisted analysis.

## Discussion

### Summary of findings

This study presents one of the first systematic evaluations of ChatGPT for qualitative analysis of clinical free-text data, adapting prompt engineering techniques to optimize performance. In the deductive coding tasks, performance varied across the 2 analytic categories.

For Category 1 (reasons for deterioration), even a minimal baseline prompt yielded substantial agreement (κ = 0.67), likely reflecting the relatively constrained and objective nature of clinically dense comments. Layering strategies like adding role, context, and few-shot provided only incremental gains, whereas stepwise instructions added little benefit.

In contrast, Category 2 started with much lower agreement (κ = 0.42), highlighting the greater variability and interpretive uncertainty of describing human actions and communication. Here, prompt strategies made a much larger difference: role and context alone improved agreement to κ = 0.73 (substantial), representing the most effective strategy. Interestingly, the full combination did not outperform the simpler role + context approach, suggesting that leaner refinements can sometimes be more effective for short, task-oriented text.

Across both categories, dialogue-based calibration step further enhanced reliability by exposing alignment gaps before scaling to the full dataset. Ultimately, with optimized prompts and calibration, both categories reached substantial agreement with human coding.

### Pragmatic strategies for prompt engineering

Drawing on empirical results and process observations, we outline pragmatic strategies for LLM-assisted qualitative coding, consisting of 2 core strategies and several practical safeguards.

The first core strategy is role and context specification, which proved to be the single most powerful refinement. By giving the model clinically grounded guardrails and reducing ambiguity in task framing, this approach consistently improved alignment with human coding. The second is calibration through pulse-checks, in which a dialogue-based review of a subsample surfaced systematic misalignments early and enhanced reliability before scaling to the full dataset.

In addition to these core strategies, we identified several safeguards that strengthen stability and reproducibility. First, formatting inputs with consistent separators (eg, date stamps) and requiring structured outputs (eg, table) reduced run-together text and post-processing errors. Next, interface effects emerged as critical: performance was notably higher when comments were pasted as plain text directly into the chat compared with when they were uploaded as spreadsheets, where the model often defaulted to keyword matching (κ ≈ 0.6-0.7 vs 0.2-0.4; see Table S7). This underscores how input modality and pre-processing can introduce hidden variability. Finally, instruction-enhancement techniques such as few-shot and stepwise prompting provided added value in some cases, though their impact was context dependent.

These strategies do not introduce novel prompting techniques but rather synthesize and adapt established methods in a way that is pragmatically tailored to clinical free-text analysis. It highlights which elements were most impactful in our setting and offers a structured reference for future researchers working with similar data.

### Implications for research and practice

Our findings indicate that LLMs can serve as a reliable partner in large-scale qualitative coding of clinical free-text data, but only when embedded within structured and safeguarded workflows. In clinical informatics, this enables scalable analysis of operational text (eg, BPA comments) for system-level learning. From a methodological standpoint, prompt refinement can play a role analogous to codebook calibration among human coders, and brief pulse-checks offer a feasible quality gate before full deployment. Practically, humans should remain in the loop to calibrate instructions and adjudicate edge cases; the goal is to enhance efficiency and extend analytical coverage, not to replace expert judgment.

### Limitations and ethical considerations

A key limitation of this study is the relatively small dataset, consisting of 778 free-text entries. While sufficient for exploratory analysis, this sample size may limit the generalizability of findings, particularly when applied to larger or more diverse datasets. In addition, the study’s focus on clinical BPA data may further restrict generalizability to datasets with varying structures, subjectivity, or linguistic complexity. Additionally, deductive thematic analysis was restricted to assigning a single best-fitting theme per comment, prioritizing inter-rater reliability over thematic richness. Future research should explore multi-label coding approaches to balance analytical depth and classification accuracy.

The use of LLMs like ChatGPT in qualitative research raises ethical concerns that require careful consideration. Two key issues stand out: First, any data uploaded to ChatGPT contributes to future model training,^24^ posing concerns about data confidentiality and unintended information exposure. While no PHI was included in this study, researchers must remain aware of broader implications regarding data security. This risk could be mitigated if institutions provide researchers with access to high-performing secure LLMs that do not retain user data for model training. Second, AI-assisted research raises questions regarding authorship and intellectual contributions.^25^ As journals and institutions have varying policies on AI, researchers must adhere to publication guidelines and transparently acknowledge AI involvement to uphold academic integrity.

### Future directions

Beyond the study-specific limitations, our findings point several avenues for future research. First, there is a need to develop standardized best practices for prompt engineering in clinical informatics. While our study offers pragmatic strategies, future work could formalize these into a handbook or set of guidelines to strengthen reproducibility, transparency, and rigor in LLM-assisted qualitative research. Second, comparative studies across domains, datasets, and LLM platforms will be essential to assess generalizability and identify context-specific adaptations. Finally, while this study demonstrated the value of pulse-checks as a human-in-the-loop calibration step, future research should more systematically evaluate alternative models of human-AI collaboration. Beyond simple model-human comparisons, it will be important to test workflows such as AI-first coding with human auditing, fully interactive refinement cycles, or hybrid approaches. Rigorous evaluations of these models—measuring not only agreement but also efficiency gains (time/cost savings), error detection, interpretability, and downstream analytical validity—will be critical. Together, such investigations could provide a blueprint for integrating LLMs into qualitative research pipelines in a way that enhances productivity, preserves integrity, and aligns with the standards of reproducible science.

## Conclusion

The study demonstrates the feasibility of using ChatGPT for qualitative analysis of BPA free-text comments, showing that with structured prompt design and calibration, the model can achieve substantial agreement with human coding. More importantly, our work advances a pragmatic methodology for prompt engineering in clinical text—role and context specification, calibration steps, and safeguards—to enhance reliability and reproducibility in LLM-assisted analysis. As large-scale free-text datasets increasingly exceed the capacity of manual review, such approaches can help unlock insights while preserving interpretive depth. Future research should continue to evaluate their utility across diverse datasets and explore best practices that balance efficiency with rigor. As LLMs become more widely adopted, responsible integration with human expertise will be essential for their safe and effective use in qualitative research.

## Supplementary Material

ooaf126_Supplementary_Data

## Data Availability

The data underlying this article will be shared on reasonable request to the corresponding author.
