# Supplementary material for: Leveraging ChatGPT for thematic analysis of medical best practice advisory data
Source: JAMIA Open. 2025 Oct 27;8(5):ooaf126. doi: 10.1093/jamiaopen/ooaf126 (PMC12757007; doi:10.1093/jamiaopen/ooaf126)
Supplement: ooaf126_Supplementary_Data [file ooaf126_supplementary_data.docx]

**Supplemental Appendix**

**eFigure 1.** Thematic analysis result for Category 1 (reasons for deterioration).

**
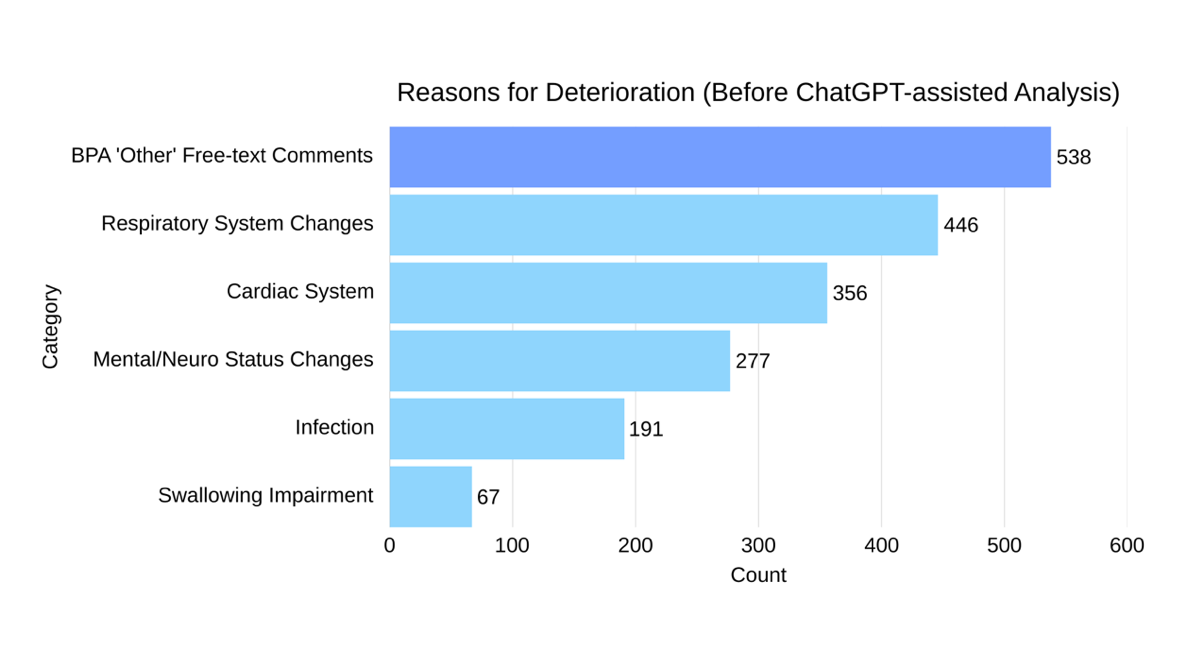
**

**
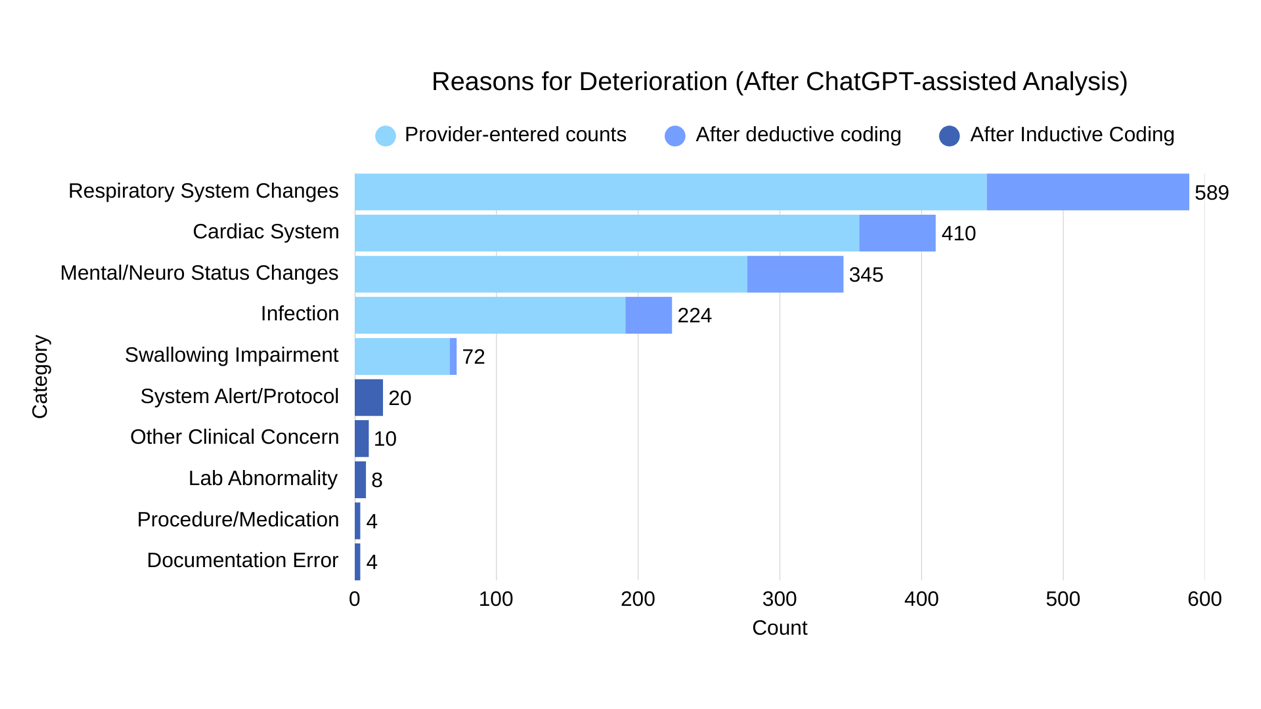
**

The top panel displays counts from the original BPA data prior to free-text analysis, while the bottom panel shows the updated distributions after deductive and inductive coding. Most comments mapped to predefined categories, while inductive analysis added themes such as System Alert/Protocol and Lab Abnormality, although these were less frequent than major drivers like respiratory or cardiac deterioration.

**eFigure 2.** Thematic analysis result for Category 2 (care team actions)**
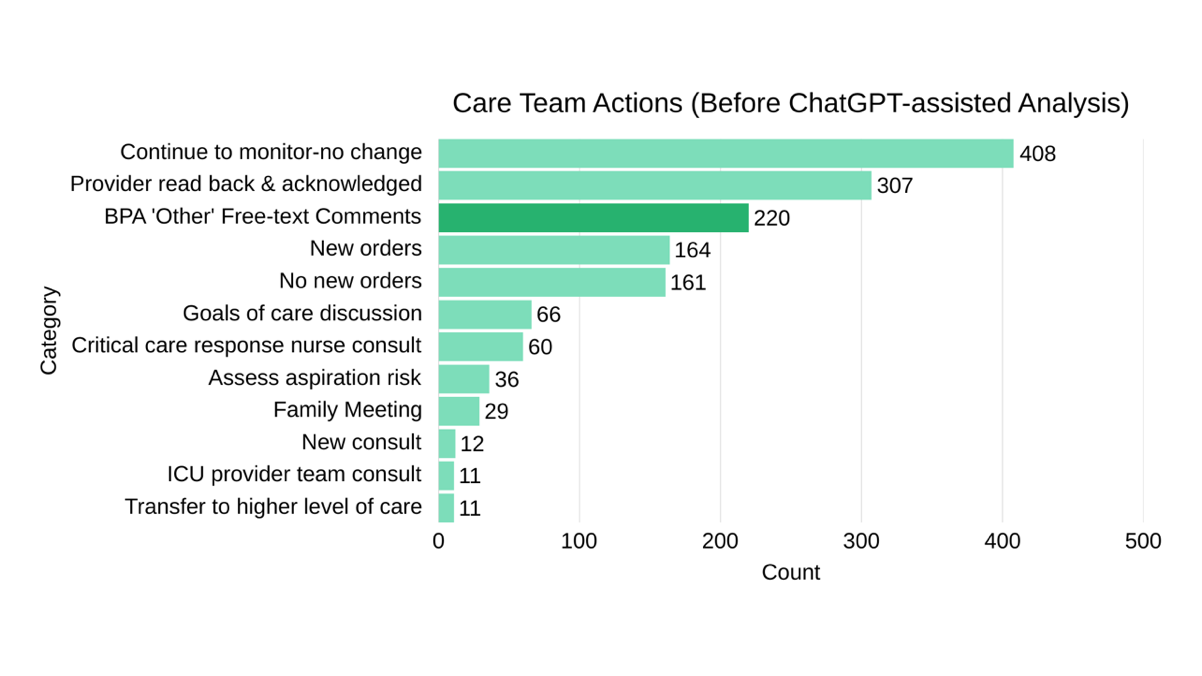
**

**
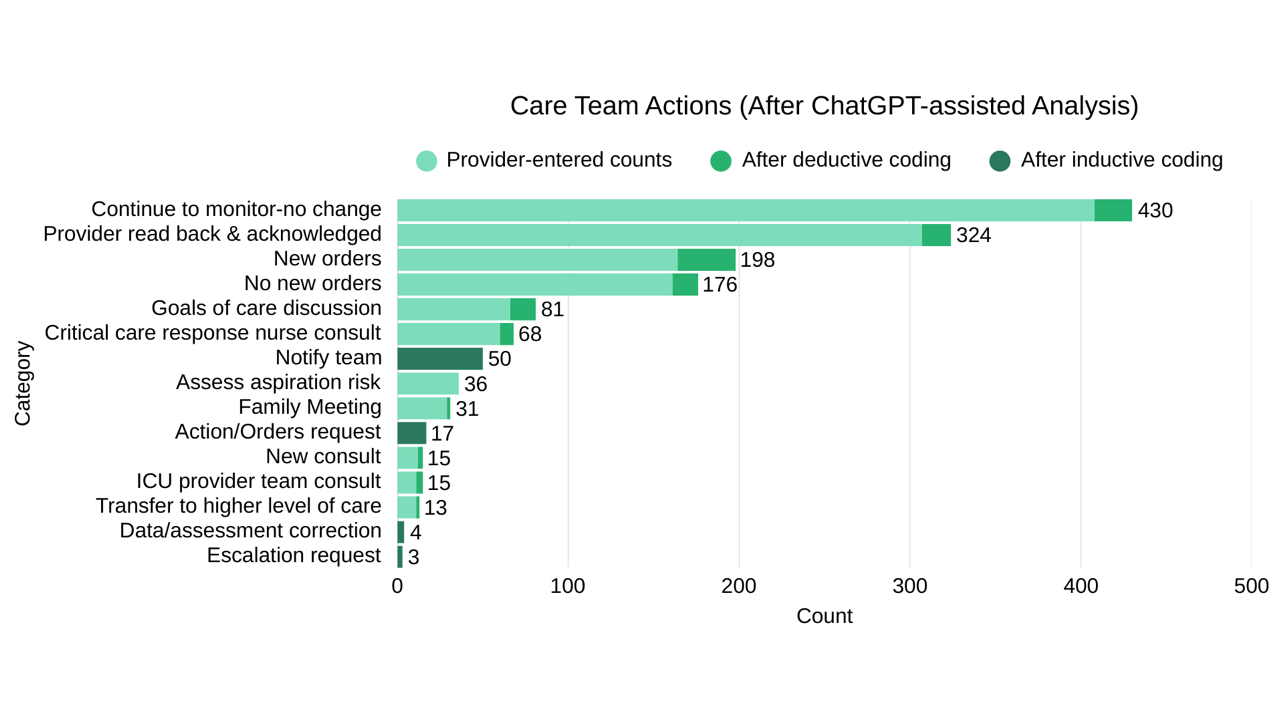
**

The top panel shows the original BPA data before free-text analysis, and the bottom panel displays the updated distributions after deductive and inductive coding. Deductive coding reassigned some of “Other” comments into predefined categories and inductive analysis generated four new themes, with Notify Team and Action/Orders Request most frequent; however, Continue to Monitor remained the overall most common action.

**eTable 1.** Codebook for Category 1 (reasons for deterioration).

| Code | Definition |
| --- | --- |
| Cardiac System | Descriptions related to the function and condition of the heart and circulatory system, excluding arrhythmia. |
| Infection | Any indication of bacterial, viral, fungal, or other types of infections including symptoms, confirmed diagnoses, and relevant lab test results. |
| Mental/Neuro Status Changes | Any observation of changes in the patient's mental or neurological status, including alterations in consciousness, confusion, delirium, seizures, and other neurological symptoms. |
| Respiratory System Changes | Any mention of changes in the patient's respiratory function, including breathing difficulties, oxygen saturation levels, respiratory distress, and use of interventions like supplemental oxygen or mechanical ventilation. |
| Swallowing Impairment | Any indication of difficulty swallowing (dysphagia) that may put the patient at risk for aspiration or other complications. |
| Other | Any comment that describes conditions, complications, or observations that do not clearly fit under the existing predefined themes. |

**eTable 2.**  Codebook for Category 2 (care team actions).

| Code | Definition |
| --- | --- |
| Assess Aspiration Risk | Actions or considerations related to evaluating the patient’s risk of aspiration, such as swallowing assessments or interventions to prevent aspiration. |
| Continue to Monitor - No Change | Decision to maintain the current treatment or monitoring plan without making changes. |
| Critical Care Response Nurse Consult | Involvement of a critical care response nurse to assess or intervene in the patient’s care. |
| Family Meeting | Documentation of a discussion with the patient’s family regarding their condition, treatment plan, or prognosis. |
| Goals of Care Discussion | Discussions focused on the patient’s goals, preferences, and values regarding their care and treatment options. |
| ICU Provider Team Consult | Involvement of the ICU provider team for evaluation or management of the patient. |
| New Consult | Initiating a consultation with a new specialty or service. |
| New Orders | Introduction of new orders for diagnostics, treatment, or interventions as a response to the alert. |
| No New Orders | Explicit documentation that no new orders were placed as a result of the alert. |
| Provider Read Back & Acknowledged | Confirmation that the provider has reviewed and acknowledged the alert, often with a documented verbal or written "read back." |
| Transfer to Higher Level of Care | Decision to transfer the patient to a higher acuity unit, such as the ICU or step-down unit. |
| Other | Any response or documentation that does not fit into the predefined categories. |

**eTable 3.** Final prompt for deductive coding.

| **Category 1: Reasons for deterioration [baseline + role + context + stepwise + few-shot]**  “You are a clinical researcher trained in qualitative analysis of electronic health record (EHR) free-text comments. You are working on a hospital project analyzing provider documentation of patient deterioration.  Classify each comment into one of the following categories representing reasons for deterioration: [Cardiac, Infection, Mental/Neuro, Respiratory, Swallowing impairment, Other].  Follow these steps for each comment:  1. Read the comment carefully.  2. Extract clinical cues (keywords or signs that point to a system).  3. Map cues to the categories from list above and determine which system is the most likely or immediate cause of deterioration. Consider the overall clinical context – don’t just map based on a single keyword.  4. Decide on the single best-fitting category. Use “Other” only if no other category fits at all.  5. Output the result in a two-column table: [Comment] \| [Category]”  Here are some examples:  Comment: “Low BP, HR 140, O2 sat stable” → Category: Cardiac  Comment: “Fever 100.4, WBC 17, possible pneumonia” → Category: Infection  Borderline examples:  Comment: “RR 28, O2 sat 92, fever 100” → Category: Respiratory  Comment: “GCS 8, O2 sat 96 on 2L NC” → Category: Mental/Neuro  “Other” examples:  Comment: “Hb 6.2, transfusion ordered” → Category: Other  Comment: “Awaiting CT scan” → Category: Other”  **Category 2: Care team actions [baseline + role + context]**  “You are a clinical researcher trained in qualitative coding of electronic health record (EHR) free-text comments. You are working on a hospital project analyzing provider documentation of patient deterioration.  Classify provider comments into one of the following categories representing care team actions: [Assess aspiration risk, Continue to monitor, Critical care response nurse consult, Family meeting, Goals of care discussion, ICU provider team consult, New consult, New orders, No new orders, Provider read back, Transfer to higher level of care, Other].  Apply clinical judgment to select the most appropriate category that best represents the provider’s documented action. Output results in a two-column table: [Comment] \| [Category].” |
| --- |

**eTable 4.** Example of dialogue-based refinement (calibration step).

| **Human**: [initial prompt: “You are a clinical researcher trained in qualitative analysis…”]  **ChatGPT**: [displays initial coding results]  **Human**: Great! Before we move on to the next set of comments, I'd like to compare our coding results to identify any discordant cases where we've coded the same comment differently. This will help ensure we're aligned in our approach. How does that sound?  **ChatGPT**: That sounds like a very solid plan—I agree completely. Comparing for **discordant cases** will help us calibrate coding rules, refine how we interpret borderline situations, and prevent systematic drift as we move through larger sets.  **Human**: Here are the discordant cases. Can you provide your reasoning/rationale behind your coding decision? [provides discordant cases]  **ChatGPT**: Perfect — thanks for pulling those out. Let me walk through each discordant case and explain why I coded it the way I did. I’ll focus on **clinical cues → mapping → rationale** so you can see my decision-making process. [provides rationale]  **Human**: [explains own rationale; accepts if aligned, suggests reclassification if not]  **ChatGPT**: This is great—we’re really honing in on the nuances of how to apply the coding scheme consistently. Let me respond to each of your suggested reclassifications, with my perspective:  [repeat this process as needed]  **ChatGPT**: I’m aligned with: [lists reclassified cases]. Would you like me to update the **Excel file** with these consensus reclassifications so we have a clean version going forward? |
| --- |

**eTable 5.** Prompt used for inductive coding.

| “You are a clinical researcher trained in qualitative analysis.  Previously, we performed deductive coding of clinical free-text comments into the following categories:  (Category 1) [Cardiac, Infection, Mental/Neuro, Respiratory, Swallowing impairment, Other]. (Category 2) [Assess aspiration risk, Continue to monitor - no change, Critical care response nurse consult, Family meeting, Goals of care discussion, ICU team consult, New consult, New orders, No new orders, Provider read back, Transfer to higher level of care, Other].  The following comments were originally coded as "Other." Your task is to perform inductive thematic coding on these "Other" comments to discover new, meaningful themes.  Instructions:  1. (Category 1) Read each comment carefully and identify the underlying reason why the deterioration alert was triggered; (Category 2) Read each comment carefully focusing on the care team’s actions in response to the patient deterioration alert in the EHR.  2. Generate new themes inductively from the data.  3. Each comment should be assigned exactly one theme.  4. Theme names should be short (1–3 words). Aim to generate no more than 5 themes in total.  4. Provide a brief definition (1–2 sentences) for each theme you create.  5. At the end, output:  (1) a list of all new themes you identified with their definitions  (2) a table mapping each original comment to its assigned theme.  Here are the clinical notes to code: [Insert comments here]” |
| --- |

*Note.* Inductive coding prompts were not iteratively optimized; instead, we adapted effective deductive strategies (role specification, context, and stepwise instructions) into a single pragmatic prompt for theme generation.

**eTable 6.** Inductive coding results (human-model comparison)

| **Category 1** (reasons for deterioration, n = 46) | | | |
| --- | --- | --- | --- |
| Human Coder | | ChatGPT | |
| Theme | Count | Theme | Count |
| Non-deterioration/Protocol  Sub-theme 1: No deterioration  Sub-theme 2: Patient Factors | 26 | System Alert/Protocol | 20 |
| Physiological Drivers  Sub-theme 1: Hematologic  Sub-theme 2: Lab Abnormality  Sub-theme 3: Metabolic  Sub-theme 4: Temperature | 16 | Other Clinical Concern | 10 |
|  |  | Lab Abnormality | 8 |
| Technical/Contextual Factors  Sub-theme 1: Error  Sub-theme 2: Procedure | 4 | Procedure/Medication-related | 4 |
|  |  | Data/Documentation Error | 4 |
| **Category 2** (care team actions, n = 74) | | | |
| Human Coder | | ChatGPT | |
| Theme | Count | Theme | Count |
| Inform Clinician | 57 | Notify Team | 50 |
| Guidance/Plan of Care Request | 11 | Action/Orders Request | 17 |
| Order Request | 2 |  |  |
| Escalation Request | 2 | Escalation/Disposition Request | 3 |
| Error Correction | 2 | Data/Assessment Correction | 4 |

*Note.* For Category 1, 54 comments were coded as “Other,” but 8 were excluded from inductive analysis because the human coder had assigned them to predefined categories. For Category 2, 87 comments were coded as “Other,” with 13 excluded for the same reason.

**eTable 7.** Iterative prompt refinement on the Category 1 subsample (n = 70, spreadsheet upload).

| Iteration | Prompt Strategy | Matched N (%) | Cohen’s κ (range) | Agreement |
| --- | --- | --- | --- | --- |
| I0 | Baseline | 32/70 (45.7%) | 0.28 (0.24-0.36) | Fair |
| I1 | Baseline + [Role + Context] | 34/70 (48.6%) | 0.31 (0.24-0.43) | Fair |
| I2 | Stepwise instructions | 43/70 (61.4%) | 0.43 (0.40-0.47) | Moderate |
| I3 | Stepwise + [Role + Context] | 37/70 (52.9%) | 0.37 (0.29-0.50) | Fair |
| I4 | Few-shot prompting | 29/70 (41.4%) | 0.24 (0.21-0.27) | Fair |
| I5 | Few-shot + Stepwise | 24/70 (34.3%) | 0.17 (0.11-0.26) | Slight |

*Note.* Agreement with human coders was substantially lower when comments were uploaded via spreadsheet than when they were entered through direct copy–paste (Main Document Table 2), even within the same subsample.
